# *Purpureocillium lilacinum* SBF054: Endophytic in *Phaseolus vulgaris*, *Glycine max*, and *Helianthus annuus*; Antagonistic to *Rhizoctonia solani*; and Virulent to *Euschistus heros*

**DOI:** 10.3390/microorganisms12061100

**Published:** 2024-05-29

**Authors:** Flávia Melo Moreira, Túlio Iglésias Machado, Caio Augusto Rosado Torres, Hebert Ribeiro de Souza, Matheus Felipe Celestino, Marco Antônio Silva, Giovana Cidade Gomes, Breno Beda dos Reis Cunha, Pedro de Luca Buffon dos Santos, Magno Rodrigues de Carvalho Filho, Marcelo Tavares de Castro, Rose Gomes Monnerat

**Affiliations:** 1 SoluScience, SoluBio Tecnologias Agrícolas, Brasília 70632-300, Brazil; caioartt@gmail.com (C.A.R.T.); hebert.souza@solubio.agr.br (H.R.d.S.); matheus.celestino@solubio.agr.br (M.F.C.); marco.silva@solubio.agr.br (M.A.S.); giovana.gomes@solubio.agr.br (G.C.G.); breno.cunha@solubio.agr.br (B.B.d.R.C.); pedro.buffon@solubio.agr.br (P.d.L.B.d.S.); magnorcf@gmail.com (M.R.d.C.F.); marceloengflorestal@gmail.com (M.T.d.C.); rosemonnerat@gmail.com (R.G.M.); 2 Department of Agronomy, Purdue University, West Lafayette, IN 47907, USA; tulioiglesias96@gmail.com

**Keywords:** blastospores, endophytic colonization, extracellular enzymes, entomopathogenic fungus

## Abstract

Microorganisms with multiple ecological functions can be a useful biotechnological resource in integrated pest- and disease-management programs. This work aimed to investigate the potential endophytic and virulent effects of a strain of *Purpureocillium lilacinum* on organic cultivation in Brazil. Specifically, the strain’s ability to establish itself as an endophyte in common bean, soybean, and sunflower plants when inoculated via seed was evaluated. Furthermore, its antifungal activity against phytopathogens and its pathogenicity and virulence against insects of the order Lepidoptera, Coleoptera, and Hemiptera were evaluated. Furthermore, the strain was evaluated for its biochemical and physiological characteristics. For virulence bioassays, the experiments were conducted under a factorial scheme (2 × 3), with the following factors: (a) fungal inoculation and control without inoculum and (b) types of inocula (blastospores, aerial conidia, and metabolites). The treatments were sprayed on insect species at different stages of development. In summary, it was found that the SBF054 strain endophytically colonized the common bean, with partial recovery from the root tissues of soybean and sunflower plants, 30 days after inoculation; suppressed 86% of *Rhizoctonia solani* mycelial growth in an in vitro assay; and controlled eggs, nymphs, and *Euschistus heros* adults. These multifunctional abilities are mainly attributed to the strain’s mechanisms of producing metabolites, such as organic acids, soluble nutrients, and hydrolytic enzymes.

## 1. Introduction

Brazilian agroindustry is one of the few segments that has presented a positive economic balance over recent years. In 2022, agricultural revenue was responsible for 68.53% of the GPV (gross value of agricultural production), reaching values around BRL 1 trillion, classified as the second-highest value in the last 34 years [1]. The gradual evolution of revenue in the agricultural sector is due to the innovations and agricultural practices implemented, such as genetic improvement programs, the use of high-quality seeds, and chemical inputs [2].

Important crops in the Brazilian scenario, such as soybeans, beans, and sunflowers, have benefited from these practices. In the 2021/2022 harvest, these crops increased their production by 0.56%, 3.33%, and 13.53%, respectively, concerning the 2020/2021 harvest [3]. Concomitant to productivity, the use of chemical inputs continues to increase. However, the indiscriminate use of fertilizers and synthetic inputs can significantly harm the physical, chemical, and biological properties of the soil and, therefore, the quality of future products. Furthermore, resistant pathogens are selected in the field, reducing product efficiency [4,5].

In this scenario, using microorganisms beneficial to agriculture has become an increasingly frequent management strategy to reduce the amount of chemical inputs used without losses in productivity [6]. When established in the environment, these microorganisms can act as a biocontrol agent for pests and diseases, growth promoters, and resistance inducers, as well as increase fertilization and promote soil correction [6,7,8,9,10]. One of the species used to formulate biological products based on microorganisms is the fungus *Purpureocillium lilacinum* (Thom.) (=*Paecilomyces lilacinus*) [11].

*Purpureocillium lilacinum* is a cosmopolitan fungus that can be isolated from nematodes, insects, soil, and the rhizospheres of plants [12,13]. It is considered a biocontrol agent for plant-parasitic nematodes, especially the species *Meloidogyne incognita* and *Tylenchulus semipenetrans* [14,15]. In addition to its ability to biocontrol nematodes, this species parasitizes insect pests, including the citrus blackfly (*Aleurocanthus woglumi*), pepper thrips (*Scirtothrips dorsalis*), tomato leafminer (*Tuta absoluta*), whitefly (*Bemisia tabaci*), earworm (*Helicoverpa zea*), and leaf-cutter ant (*Acromyrmex lundii*) [8,10,16,17,18,19]. *Purpureocillium lilacinum* contains endophytic strains that bring benefits to the plant, both for promoting growth and inducing resistance to herbivorous insects [7,16,20].

When endophytic, *P. lilacinum* resides intercellularly in plant tissues and does not cause visible damage or morphological changes. However, reports on the use of this species to promote plant growth are rare and limited to tomato cultivation [7,20,21]. A better understanding of the endophytic capacity of the strain associated with pathogenicity to different groups of insects will be useful for establishing a sustainable environment in the agricultural production system.

In addition to the ability to parasitize insects and colonize plants, strains of *P. lilacinum* showed mycoparasitism on phytopathogenic fungi, including *Sclerotinia sclerotiorum* and *Phytophthora infestans* [22,23]. The pathogenicity of this fungus is related to the production of bioactive secondary metabolites, such as antibiotic leucinostatins called paecilotoxins, and extracellular enzymes (chitinases, proteases, and lipases) [13,23,24]. In this context, the inoculation of beneficial microorganisms that act on multiple fronts, such as the secretion of antibiotics and cell-wall degradation enzymes, can be considered a useful technological resource for farmers through preventive and/or curative actions against pests and diseases [6,9].

Considering the growing need for efficient strategies in the management of pests and diseases in the agricultural system, the present work aimed to investigate the potential endophytic and virulent effect of a strain of *P. lilacinum* originating from organic cultivation in the Federal District, Brazil. Specifically, the strain’s ability to (1) establish itself as an endophyte in common bean, soybean, and sunflower plants when inoculated via seed; (2) antagonize phytopathogens; and (3) its pathogenicity and virulence in insects of the order Lepidoptera, Coleoptera, and Hemiptera under laboratory conditions were evaluated.

## 2. Material and Methods

### 2.1. Origin of the Fungus

Soil samples were collected in 8 subareas of an organic horticulture system, located in Brasília/DF (15°37′55″ S, 47°55′21″ W, altitude 1171 m), Brazil. The subareas differed by the crops and management adopted. In each subarea, 20 simple samples of approximately 100 g of soil were taken from the surface layer (0 to 10 cm deep) and between the plants, collected randomly and in a zigzag pattern. In the end, they were homogenized, forming a sample composed of subareas [25].

In the laboratory, soil samples were homogenized in saline solution (0.9% NaCl), in a 1:10 (*v*:*v*) ratio, and then used for serial dilutions followed by plating on selective Sabouraud dextrose agar media (HiMedia Laboratories, Kennett Square, PA, USA) to isolate the entomopathogenic fungi [26]. The plates were kept in a Biological Oxygen Demand (B.O.D) incubator (415D, Ethik Technology, São Paulo, Brazil) for 10 days at 26 °C and a 12 h photoperiod.

### 2.2. Morphological, Molecular, and Biochemical Characterization of the Strain

The fungal colony was purified in PDA medium (potato dextrose agar) (HiMedia Laboratories, USA) supplemented with 0.5 g L^−1^ of chloramphenicol (INLAB, São Luís, Brazil) and then identified through morphological characteristics and reproductive cells [11,27]. The strain of interest was then stored at −18 °C for later use. The fungus, deposited in the Microorganism Collection of SoluBio Tecnologias Agrícolas, was registered with the identification code SBF054.

After morphological analysis, the strain was subjected to molecular analysis for species-level identification. To guarantee purity, monosporic culture was used to extract genomic DNA. The sequence obtained was analyzed using the Mash tool [28]. In addition to morphological and genetic characterization, the strain was analyzed for its ability to synthesize siderophores, organic acids, and extracellular enzymes, such as chitinase, protease, lipase, cellulase, amylase, phosphatase, and solubilize phosphorus and potassium [6,9,29,30,31,32,33,34,35].

The extracellular activity of lytic enzymes, such as chitinase, protease, and lipase, were measured every other day for 10 days and evaluated using the degradation index [ID = A/B], where A is the diameter of the colony and B is the total diameter (colony + degradation halo) [35]. Degradation efficiency is evaluated according to the scale proposed by Price in 1982 [36], in which (ID = 1), the isolates cannot synthesize the enzyme, (1 > ID > 0.69) low; (0.69 > ID > 0.3) high; (ID < 0.3) very high synthesis. The tests were conducted twice with five repetitions.

### 2.3. Production of Aerial Conidia, Blastospores, and Metabolites

Aerial conidia were produced in Petri dishes containing PDA medium supplemented with chloramphenicol at a temperature of 26 °C and a 12 h photoperiod for 10 days in an incubator. After this period, these propagules were removed from the Petri dishes by scraping the mycelium and homogenizing in a Tween 80 solution (0.05%), with the proportion of one plate having 10 mL of diluent, and subsequent measurement in a Neubauer chamber under an optical microscope (×40). The production of aerial conidia was carried out in triplicate, and their germination rates were evaluated as described by Ansari and Butt in 2011 [37] and always exceeded 90%.

Blastospore production was carried out through submerged liquid fermentation (FLS) in an SM culture medium (40 g L^−1^, pH = 6.00), which is a product formulated by the company SoluBio Tecnologias Agrícolas, Brazil. To quantify the blastospores produced by *P. lilacinum*, SBF054 strain, a fungal suspension was added to Erlenmeyer flasks with a capacity of 250 mL and containing 90 mL of the SM culture medium and 10 mL of the fungal suspension (1 × 10^7^ conidia mL^−1^). The preparation of this culture medium followed the manufacturer’s description. The flasks were kept under agitation at 200 rpm and a constant temperature of 26 °C in the dark for 72 h.

The performance of the strain in the SM culture medium was evaluated in three replications, and the assay was repeated three times. After 72 h of cultivation, the number of blastospores was estimated using the Neubauer chamber; the number of microsclerotia (MS) on a glass slide was visualized using a light microscope (Axiolab5, Zeiss, Oberkochen, Germany) [38]. The count of viable propagules was obtained by plating the samples on PDA supplemented with chloramphenicol. The results were expressed in blastospores mL^−1^, MS mL^−1^, and CFU mL^−1^, respectively. Blastospore germination rates were evaluated following the protocol described by Corrêa in 2020 [39] and exceeded 90%.

For the production of metabolites, conditions similar to blastospores were used in terms of culture medium and environmental conditions. However, to obtain the maximum metabolites, liquid fermentation lasted seven days. Subsequently, the samples were filtered and centrifuged (5000 rpm), ensuring the removal of the propagules produced.

### 2.4. Endophytic Effect on Bean, Soybean, and Sunflower Plants

The in vivo experiment was conducted in a greenhouse at the SoluScience Research Center by SoluBio in Brasília, Distrito Federal, Brazil (15°46′48″ S, 47°55′45″ W, 1130 m altitude). The local climate is type Aw, Tropical with a dry season according to the Koopen and Geiger climate classification; the average annual temperature is approximately 21 °C. The experimental design used to evaluate the performance of the plants inoculated, via seed, with *P. lilacinum* was completely randomized in three plant species and five replications.

Common beans (*Phaseolus vulgaris*), soybeans (*Glycine max*), and sunflowers (*Helianthus annuus*) were purchased from local markets. The seeds were superficially disinfected with 1% sodium hypochlorite for three minutes, followed by triple washing in sterile distilled water [40]. Finally, the seeds were immersed in a conidium suspension at a concentration of 10^8^ aerial conidia mL^−1^ for 30 min and sown in a mixture of washed sand and organic fertilizer, in a ratio of 3:1 (*v*:*v*). The cultivation substrate was placed in polypropylene pots with a capacity of 300 cm^3^. Seeds homogenized in sterile distilled water were used as control.

Irrigation was carried out manually. After 30 days, the plants were segmented into roots, stems, and leaves, and the roots were washed in running water to remove all adhering material. Subsequently, the parts were disinfected in the following sequence: 70% alcohol and 0.5% sodium hypochlorite for 30 s, and triple washing in sterile distilled water. The disinfected plant segments were deposited in Petri dishes containing PDA and placed in a B.O.D incubator (415D, Ethik Technology, Brazil) at 25 °C for 10 days.

Root clarification, coloring of fungal structures, and cultivation of Koch’s Postulate were carried out to verify the endophytic colonization of the fungus in different plants according to the methodologies proposed by Phillips and Hayman in 1970 and Brundett in 1996 [9,41,42].

### 2.5. Antagonism to Phytopathogenic Fungi

A 5 mm diameter mycelium disc from the pure culture of *P. lilacinum* and another disc from the pathogenic fungus were added to opposite sides of the Petri dish (90 × 15 mm, CRAL, Cotia, SP, Brazil) containing the PDA culture medium [43]. The plates were incubated at 26 °C, with a 12 h photoperiod of 10 days. The experiment was conducted twice with five replications. *Fusarium oxysporum*, *M. phaseolina*, and *R. solani* were used in a culture pairing test. The zone of inhibition (ZI) was measured at the shortest distance between both cultures, and the inhibition of pathogen growth was calculated by the following formula:

Inhibition of mycelial growth (%) = [100 × (r1 − r2)/r1](1)

where r1 and r2 are the longest and shortest pathogen mycelial growth, respectively [43].

### 2.6. Mortality Bioassays against Insects of the Orders Lepidoptera, Coleoptera, and Hemiptera

Caterpillars and egg masses of *Spodoptera frugiperda* (Lepidoptera) were collected from corn and soybean crops and raised on an artificial diet according to the protocol [44]. The creation was carried out in the laboratory under a temperature of 25 ± 2 °C, relative humidity (RH) of 55% ± 10%, and a 12 h photophase. The *Anthonomus grandis* (Coleoptera) colony was developed in the laboratory under an artificial diet to obtain eggs, larvae, and adults, following the recommendations of Monnerat and Martins [45,46,47]. Regarding *Euschistus heros* (Hemiptera), adults were placed in plastic pots (1 L) with the lids cut in the center and with organza-type fabrics fixed to the opening to allow ventilation. To maintain bed-bug breeding, a natural diet consisting of bean pods (*P. vulgaris*) and cotton moistened with distilled water was offered to serve as a source of water and humidity [47]. Third instar larvae of *S. frugiperda*; *A. grandis* adults; eggs, 2nd instar nymphs, and *E. heros* adults were used in the present study.

From the three types of inocula (aerial conidia, blastospores, or metabolites), bioassays were carried out using airbrush equipment (Worker, Curitiba, PR, Brazil) (flow rate of 0.79 L/h), application of 1 mL of inoculum, at a concentration of 1 × 10^8^ propagules mL^−1^ on a plate containing 10 insects. For the control, a Tween 80 solution (0.05%) was sprayed on the insects. Then, the insects were separated into Petri dishes (90 × 15 mm, CRAL, Brazil) containing the respective artificial diets for each species and were kept in a B.O.D. air-conditioned chamber (TE-402 240L, TECNAL, Piracicaba, SP, Brazil) at a temperature of 26 ± 2 °C, RH: 60 ± 10%, and a 14 h photophase.

Mortality was assessed 13 to 15 days after application, depending on the insect, and the total cumulative mortality was obtained by summing the days assessed. In the end, the corrected mortality rate was calculated according to Abbott’s formula in 1952 [48]:

Corrected mortality = (T − C)/(100 − C) × 100(2)

where T = dead insects in the treatment and C = dead insects in the control.

To confirm that mortality was caused by the fungus, the dead insects were transferred to humid chambers maintained at 25 °C until confirmation of mycosis. Subsequently, the fungal colony present on the surface of the dead insect was isolated. The bioassays were conducted in a completely randomized design with four replications, where each replication contained ten insects for the caterpillar, nymph, and adult stages. Regarding eggs, 30 units were used per repetition. Virulence bioassays were repeated twice on different occasions.

### 2.7. Statistical Analysis

The data were evaluated for homoscedasticity and the normal distribution of residuals using the Cochran and Lilliefors tests, respectively. Then, analysis of variance and multiple comparisons of means were performed using the Tukey test (*p* < 0.05). Values were presented as mean ± standard error, with statistically significant differences indicated by different letters. Data on insect pest mortality and mycosis parameters were subjected to a principal component analysis (PCA) to explore relationships between variables and treatments using the Excel (2404 version number) and FactoMineR package in R software (4.4.0 version number) (R Core Team, Vienna, Austria).

## 3. Results

### 3.1. Morphological, Biochemical, and Molecular Characterization of the Strain

**Figure 1:**
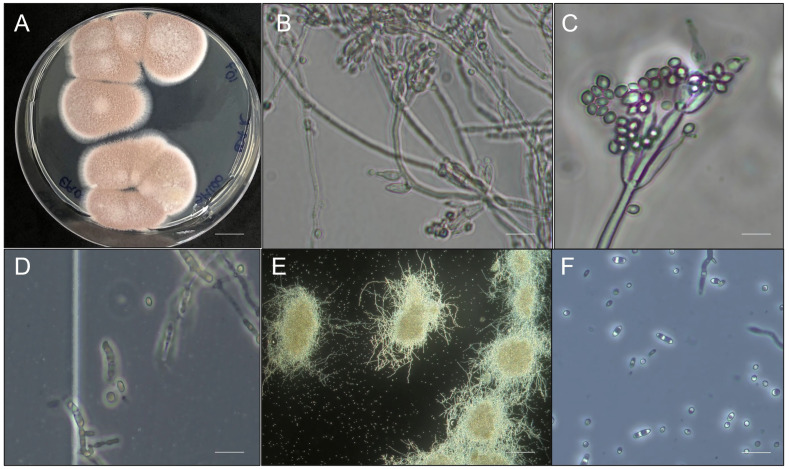
Morphological aspects of *Purpureocillium lilacinum* (SBF054) grown on a solid culture medium (potato dextrose agar): fungal colonies (scale bar: 1 cm) (**A**), hyphae and conidia (scale bar: 100 µm) (**B**), conidiophore (scale bar: 50 µm) (**C**). The propagules, blastospores (scale bar: 100 µm) (**D**), and microsclerotia (scale bar: 500 µm) (**E**) were produced under liquid fermentation after three days, and vegetative yeast cells (scale bar: 50 µm) (**F**) after seven days.

The strain was morphologically identified as a filamentous fungus belonging to the phylum Ascomycota and genus *Purpureocillium*. It presented a pink colony (reverse), a regular circular shape, and white edges. Under microscopic aspects, the hyphae are septate, and the conidiophores are whorled, supporting divergent whorls of branches and phialides. Phialides have a cylindrical basal portion with a long neck and produce unicellular and hyaline conidia (Figure 1). It produced an average of 6.0 × 10^9^ aerial conidia mL^−1^ in the PDA medium with a germination rate above 98%. When multiplied under submerged liquid fermentation in the SM medium, strain SBF054 produced 3.36 ± 1.63 × 10^8^ blastospores mL^−1^; 1.0 ± 0.71 × 10^3^ MS mL^−1^, and 1.33 ± 0.37 × 10^9^ CFU mL^−1^ after 72 h in a shaker. In addition, it produced yeast cells after seven days of fermentation (Figure 1).

**Figure 2:**
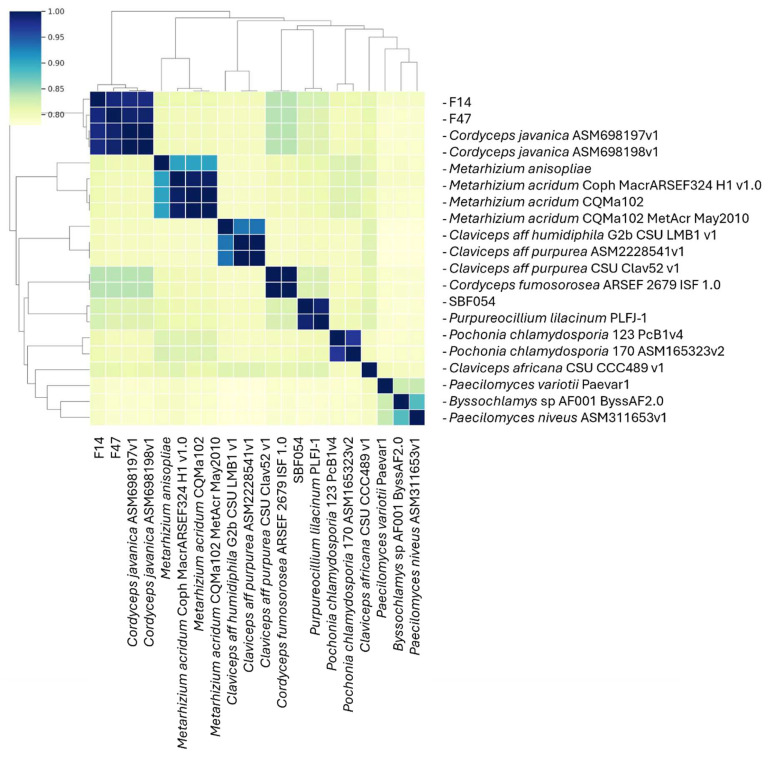
Clustermap of distance between strain SBF054 and the *Purpureocillium lilacinum* reference genome.

The isolate showed a 98.96% similarity with *P. lilacinum* and a mash of 1842 base pairs. The distance between genomes can be observed using the Clustermap (Figure 2).

The strain under study presented the following abilities: potassium solubilization, synthesis of organic acids, and hydrolytic enzymes, such as chitinase, protease, lipase, cellulase, and alkaline phosphatase (Table 1).

Among the extracellular enzymes, the protease stood out for being produced from the first evaluation, at 2 days, with a continuous degradation rate (approximately 0.77) until the tenth day. As for the lipase and chitinase enzymes, the formation of a degradation halo was recorded from the fourth and tenth day of evaluation, respectively. According to the classification proposed by Price in 1982 [36], *P. lilacinum* (strain SBF054) is considered a low producer of protease, lipase, and chitinase (Table 2).

### 3.2. Purpureocillium lilacinum Endophytically Colonizes Common Bean Plants

**Figure 3:**
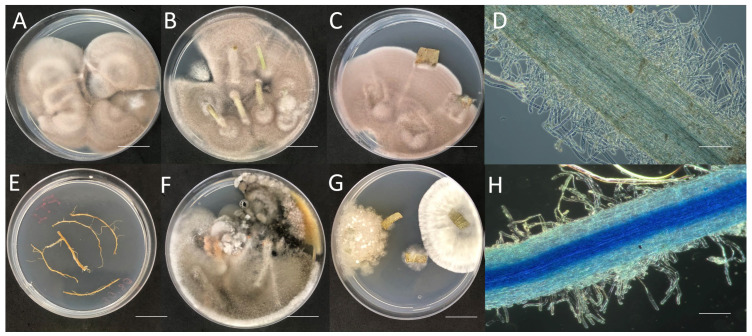
Fungal isolation in roots, stems, and leaves of young plants of *Phaseolus vulgaris* inoculated with *P. lilacinum* (scale bar: 1 cm) (**A**–**C**) and *P. vulgaris*, *G. max*, and *H. annuus* without fungal inoculation (**E**–**G**). Change in the quantity and arrangement of root hairs along the *P. vulgaris* root, with (**D**) or without inoculation with *P. lilacinum* (SBF054) (**H**). Scale bar: 1 mm in (**D**,**H**).

The percentage of endophytic colonization of SBF054 varied between agricultural species and between their parts: root, stem, and leaf. Endophytic colonization was observed in all parts of *P. vulgaris* plants through in vitro fungal isolation (Figure 3A–C). Although the effects of this strain on promoting plant growth were not evaluated, changes were observed in the number of root hairs in common bean plants, indicating a greater quantity when inoculated with the fungus (Figure 3D).

**Figure 4:**
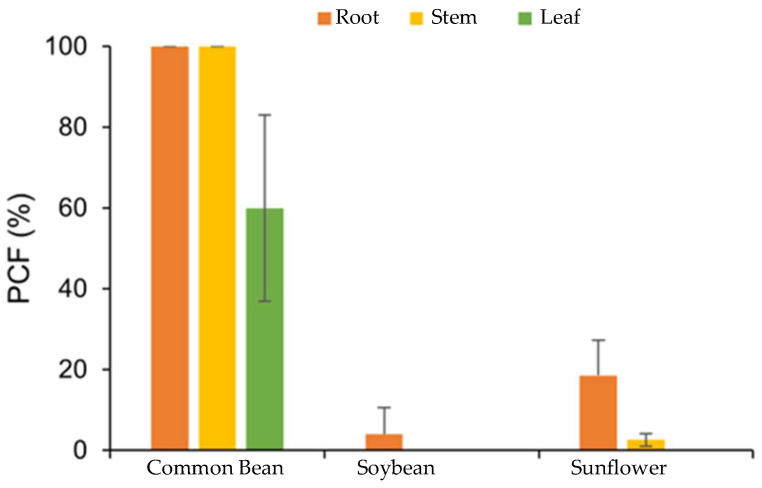
Percentage of fungal colonization (PCF) of *P. lilacinum*, strain SBF054, in agricultural crops.

The percentage of fungal colonization (PCF) exceeded the maximum colonization in the roots and stems of *P. vulgaris* plants, 30 days after fungal inoculation. There was a low percentage of colonization in the roots of *G. max* (3%) and *H. annuus* (18%). For the other parts, there was no evidence of endophytic colonization (Figure 4).

### 3.3. Antagonistic Activity against the Phytopathogen Rhizoctonia solani

**Figure 5:**
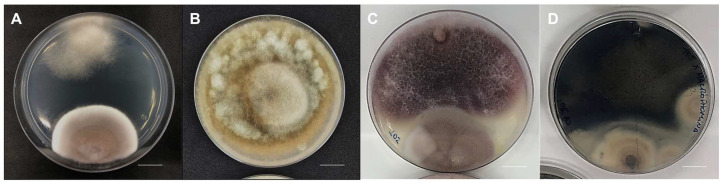
Antifungal activity of *P. lilacinum*, strain SBF054 (**A**), against *R. solani* (**A**,**B**) in vitro. The phytopathogens *Fusarium oxysporum* (**C**) and *Macrophomina phaseolina* (**D**) were not affected. Scale bar: 1 cm.

Through the results of direct pairing between the pathogen and strain SBF054, inhibition of mycelial growth of the phytopathogen *R. solani* was observed, 86.04 ± 0.13% after 10 days of incubation compared to the control. In addition, the strain produced a 2 cm inhibition zone between the cultures (Figure 5A,B). Under direct pairing with the phytopathogens *F. oxysporum* and *M. phaseolina*, their mycelium outperformed that of strain SBF054 (Figure 5C,D).

### 3.4. High Virulence of SBF054 against Euschistus heros

**Figure 6:**
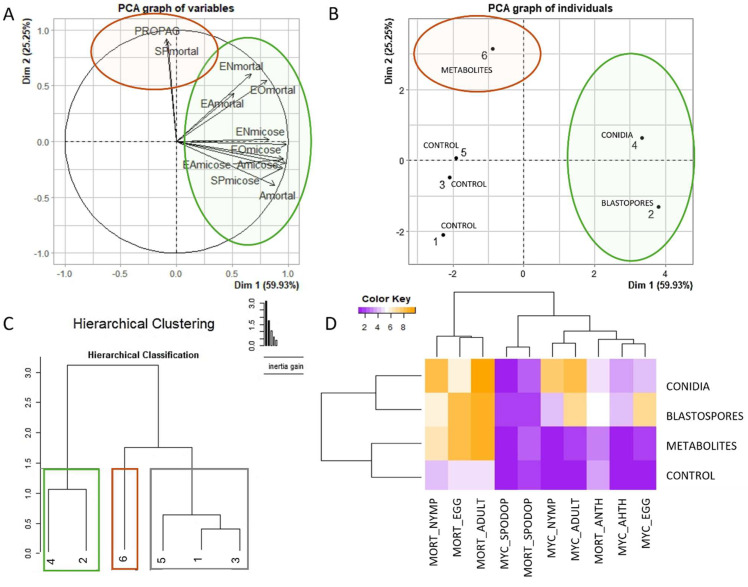
Principal component analysis (PCA) of variables (**A**) and treatments (**B**), cluster analysis (**C**), and heatmap (**D**) as a function of the virulence of the fungus *P. lilacinum* SBF054 at different stages of development [EGG, NYMP, and ADULT] of *E. heros*, caterpillars of *S. frugiperda* [SPODOP], and *A. grandis* [ANTH] adults. The variables described were related to mortality [MORT] and mycosis [MYC].

The PCA showed that the variables were positively correlated in two main components that represented 59.93% and 25.25% of the global variation, respectively. The variables were strongly affected by treatments with aerial conidia or blastospores (Figure 6A,B), forming a cluster. In addition, two distinct clusters were observed formed by treatments, control, and metabolites (Figure 6C).

A color-based heatmap clustering analysis was used to classify the pathogenicity and virulence of the *P. lilacinum* on *E. heros*, *A. grandis*, and *S. frugiperda* species. The results showed that the SBF054 strain is pathogenic and highly virulent to *E. heros*, as represented in yellow, while the untreated control showed low or no mortality, represented in purple. Furthermore, there was an interaction (*p* < 0.001) between the types of fungal inocula (aerial conidia, blastospores, and metabolites) and the developmental stages of this insect (Figure 6D).

Mortality and mycosis mortality of *S. frugiperda* and *A. grandis* insects treated with SBF054 did not differ from those obtained with the control (Figure 6D).

**Figure 7:**
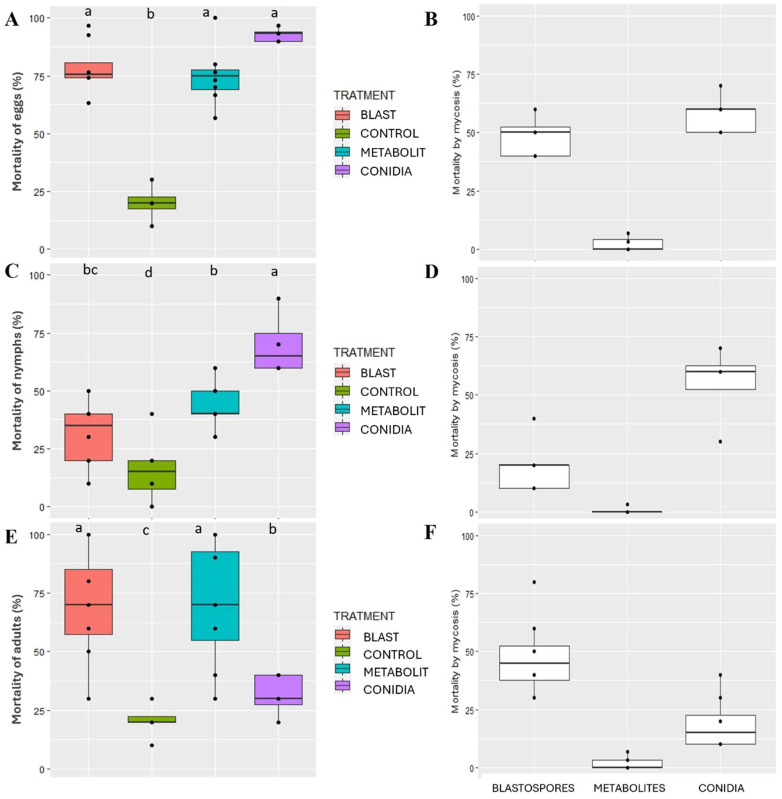
Total mortality of eggs (**A**), nymphs (**C**,**E**) *E. heros* adults, and mortality due to mycosis in the respective stages of development (**B**,**D**,**F**) after 13 days of treatment with the different inocula *P. lilacinum*, strain SBF054. Medians followed by the same letters do not differ according to the Tukey test (*p* < 0.05).

Thirteen days after SBF054 treatment, total cumulative mortality and mycosis mortality varied between inoculum types and the *E. heros* developmental stages. For eggs, there was no significant difference (*p* > 0.05) between the inocula in terms of total mortality, with a high percentage of approximately 80% being recorded. Furthermore, mortality rates from mycosis of between 50–60% were observed using blastospores or aerial conidia as inocula, respectively. Although treatment with metabolites did not promote mycosis, due to the absence of infective propagules, the egg mortality was considered high (approximately 75%) and similar to other fungal treatments (Figure 7A,B).

Regarding the nymph stage, the maximum cumulative mortality (65%) was obtained with the treatment using aerial conidia, followed by treatment with fungal metabolites (40%). When nymphs were treated with blastopores, the percentage of mortality was low, at 30%. However, the maximum mortality caused by ringworm (60%) was also recorded in second instar nymphs treated with the aerial conidia of *P. lilacinum* (Figure 7C,D).

The application of blastospores or metabolites to *E. heros* adults resulted in maximum total cumulative mortality (approximately 70%), with no statistical difference (*p* > 0.05) between treatments. When aerial conidia were used as the inoculum, only 30% mortality was observed. This same trend was observed for the variable mortality due to mycosis, in which blastospores induced greater mortality than aerial conidia (Figure 7E,F).

## 4. Discussion

The *Purpureocillium lilacinum* strain SBF054 was isolated from the soil of an organic system located in the central region of the Brazilian Cerrado. Notably, in soils under organic cultivation, cultural practices stimulate the transformation and enzymatic/biochemical activity of microbial communities, shaping soil functional diversity [49]. In this scenario, the isolation of fungi with multiple ecological functions is not uncommon. In the present study, this strain showed the ability to solubilize potassium and synthesize organic acids and enzymes that have insecticidal and antifungal properties at different levels (Table 1 and Table 2, Figure 5 and Figure 7). Furthermore, its ability as an endophyte of *P. vulgaris* (Figure 3 and Figure 4), an antagonist to the phytopathogen *R. solani* (Figure 5), and a virulent pathogen of *E. heros* at different stages of development (Figure 7) was verified.

In the environment, the responsiveness of plants to inoculation with rhizospheric microorganisms depends on the plant–microorganism interaction, the age and physiology of the targeted plant species, the location of the microorganism concerning the root system, and its specific mechanisms. Maximum benefits are obtained when endophytic microorganisms are used in inoculation or when they can reproduce in plant tissues [6,50]. Strain SBF054 was isolated from the internal tissues of young *Phaseolus vulgaris* plants, highlighting the high affinity between the microorganism and the host observed by the percentage of colonization and alteration in the quantity and arrangement of root hairs (Figure 3 and Figure 4). Some studies on endophytes showed they are known to infect specific hosts [9,51]; this can be a reason for the low endophytic colonization success in *G. max* and *H. annuus* plants (Figure 3 and Figure 4). Furthermore, this strain synthesizes acids, organic compounds, and enzymes related to nutrient availability, such as potassium solubilization and alkaline phosphatase (Table 1), which can contribute to plant growth. However, future studies are necessary to determine the benefits of this interaction on the morphophysiological characteristics of plants in terms of growth promotion.

Although in different colonization percentages, the SBF054 strain colonized the roots of all the plants evaluated (Figure 3), demonstrating its infective capacity and occupation in different root niches, such as tomato and cotton [16,20,21,52], in addition to eventually reducing the severity and incidence of diseases caused by phytopathogens [23], including *R. solani* (Figure 5). This phytopathogen is the causal agent of root-rot disease in several crops of economic importance, such as *P. vulgaris* and *G. max* [53]. In the present study, strain SBF054 showed high antifungal activity against *R. solani*, resulting in 86% mycelial inhibition under controlled conditions (Figure 5A). The benefit of these compatibility responses between microorganisms is considered relevant, especially because the selection of those with multiple aptitudes expands the possibilities of applications of a future biological input, not only for the biocontrol of nematodes but also for the fungi that cause diseases in plants.

In this study, the antifungal activity of *P. lilacinum* against *R. solani* was proven (Figure 5). As it is a soil fungus with antagonistic and nematophagous potential [14,15,22,23], it is understood that it survives in the soil in a saprophytic manner. One of the mechanisms frequently addressed is the synthesis of cell-wall lytic enzymes, such as chitinase, protease, lipase, and cellulase, which are products of the secondary metabolism of several microorganisms [13,23,24]. However, the production of these metabolites varies according to the strain and depends on the substrate used by the fungus for its colonization and reproduction. The SBF054 strain is capable of synthesizing the enzymes chitinase, protease, lipase, and cellulase at different intensity levels (Table 1 and Table 2), which is directly related to antibiosis, parasitism, and competition for space and nutrients (Figure 5 and Figure 7). It is accepted that this strain has great potential for use in the field for multiple functions, including the control of phytopathogens and insect pests.

*Purpureocillium lilacinum* has proven action against insects and has already been isolated and/or tested against several species of Hemiptera of medical or agricultural importance, such as the citrus black fly (*Aleurocanthus woglumi* Ashby; Hemiptera: Aleyrodidae), the kissing bug (*Triatoma infestans* Klug; Hemiptera: Reduviidae), and the whitefly (*Bemisia tabaci* Gennadius; Hemiptera: Aleyrodidae) [10,18,54]. Consistent with these findings, isolate SBF054 was highly efficient against *Euschistus heros* (Figure 6 and Figure 7), a phytophagous stink bug that causes great damage to several crops, especially soybeans during their reproductive phase [5,55]. The management of this pest is difficult, and currently, chemical pesticides have been used almost exclusively to control it [56].

The main barrier to the fungus’ penetration is the insect’s cuticle, composed of a thick layer made up of chitin, lipids, proteins, and other biomolecules. Infective propagules (e.g., aerial conidia, blastospores, and submerged conidia) can break this barrier through mechanical action and the exudation of lytic enzymes, such as chitinase, protease, and lipase [12]. The virulence of entomopathogenic fungi is related to their ability to synthesize these extracellular enzymes, which is important in the infection process, and the mechanical action of spore penetration [24,57]. These mechanisms were observed in this study, where both the isolated action of inocula, infective propagules (blastospores and aerial conidia), or metabolites (produced during seven days of fermentation) resulted in high mortality for *E. heros* (Figure 6 and Figure 7), suggesting that host penetration and infection is an isolated or combined action of enzymatic activity (Table 1 and Table 2) and mechanical pressure. Establishing a correlation between the production of these enzymes and virulence could be useful for the production of more effective mycoinsecticides [58].

The virulence of the SBF054 strain was recorded at all stages of *E. heros* development, and the type of inoculum interferes with mortality and mycosis (Figure 7). Aerial conidia are the most commonly found propagules in solid formulations of fungal-based biological products. However, some entomopathogenic fungi have a high capacity to produce other propagules (e.g., blastospores, submerged conidia, and microsclerotia) under liquid fermentation, depending on the multiplication conditions [59], as observed using an SM culture medium, a formulated product (Figure 1). Furthermore, the bioinsecticide activity of these propagules is also distinct and complex among fungi, and there are few studies on the virulence of *P. lilacinum* blastospores in insects. Most studies report the effectiveness of only aerial conidia in soft-bodied or larval-stage insects [8,10,17,19]. The results from different studies may not be directly comparable, as fungal isolates, insect populations, and experimental protocols are variable. In light of the present study, the virulence of *P. lilacinum* over *E. heros* is confirmed, and the differences in virulence between infective propagules are highlighted (Figure 6 and Figure 7). However, future studies are needed to evaluate the mechanisms involved in the virulence of different propagules with the host’s developmental stages, as well as their performance under field conditions.

## 5. Conclusions

In this study, it was found that the *P. lilacinum* strain SBF054 endophytically colonized *P. vulgaris*, with partial recovery from the root tissues of soybean and sunflower plants 30 days after inoculation of the plants, showed antagonistic activity against *R. solani*, and was efficient in controlling *E. heros* eggs, nymphs, and adults. This is mainly attributed to the strain’s mechanisms for producing metabolites, such as organic acids, soluble nutrients, and hydrolytic enzymes. Thus, the data presented here provide good perspectives for understanding the diverse abilities of the fungus in relation to disease and agricultural pest-management strategies. The data generated in this study have relevant implications for integrated pest-management programs because the selection of multifunctional strains can be a useful biotechnological resource for farmers. This would allow for its application to be both via soil, aiming to protect against phytopathogens, such as *R. solani*, and foliar, to control insects, such as *E. heros*. However, as part of the biological treatment validation sequence, in addition to laboratory tests, greenhouse and field tests must be carried out.

## Figures and Tables

**Table 1:** Biochemical and physiological characteristics of *P. lilacinum*, strain SBF054.

Characteristics	SBF054
Solubilization	
Potassium	+
Phosphorus	-
Synthesis	
Organic acids	+
Siderophores	-
Enzymes	
Chitinase	+
Protease	+
Lipase	+
Cellulase	+
Amylase	-
Alkaline phosphatase	+

+: positive result; -: negative result.

**Table 2:** Enzymatic activity of *P. lilacinum* (SBF054) as a function of days after inoculation.

Enzymes	Degradation Index				
	02 Days	04 Days	06 Days	08 Days	10 Days
Protease	0.75 ± 0.02	0.83 ± 0.01	0.80 ± 0.02	0.78 ± 0.01	0.70 ± 0.02
Lipase	1	0.97 ± 0.01	0.85 ± 0.03	0.84 ± 0.02	0.83 ± 0.02
Chitinase	1	1	1	1	0.97 ± 0.01
CV (%)	13.75	7.84	8.63	8.82	13.05

CV: coefficient of variation.

## Data Availability

The original contributions presented in the study are included in the article, further inquiries can be directed to the corresponding author.
